# Supplementary material for: Aggressive organ penetration and high vector transmissibility of epidemic dengue virus-2 Cosmopolitan genotype in a transmission mouse model
Source: PLoS Pathog. 2021 Mar 30;17(3):e1009480. doi: 10.1371/journal.ppat.1009480 (PMC8034735; doi:10.1371/journal.ppat.1009480)
Supplement: S1 Table — (DOC) [file ppat.1009480.s008.doc]

**Supplementary Table**

**S1 Table.** Primers used in this study
