# Supplementary material for: Aggressive organ penetration and high vector transmissibility of epidemic dengue virus-2 Cosmopolitan genotype in a transmission mouse model
Source: PLoS Pathog. 2021 Mar 30;17(3):e1009480. doi: 10.1371/journal.ppat.1009480 (PMC8034735; doi:10.1371/journal.ppat.1009480)
Supplement: Attachment — Submitted filename: Response to Reviewers 20210209.docx [file ppat.1009480.s009.docx]

**Response to Reviewers’ comments**

Part I - Summary

Reviewer #1: Here, the authors have produced a paper investigating DENV-2 strain-dependent pathogenicity and mosquito infection. The authors used a combination of experiments that relied on both mosquito inoculation and natural mosquito feeding to characterize a new strain of DENV-2 detected during an outbreak in Taiwan in 2015. Using chimeric viruses they were able to determine that both enhanced mosquito infection and enhanced mouse virulence mapped principally to prM and E. While these results are interesting, I believe there are several areas that need to be addressed to facilitate understanding by the reader and a few scientific issues that need to be addressed. Critically, there are no line numbers, which makes it difficult to call out specific areas that need improvement. Please include line numbers. Specific points follow.

**Response:**

We appreciate all the reviewers’ critics which have greatly improved our manuscript. The line numbers have been included in the manuscript.

Reviewer #2: In this manuscript, the authors examine the virulence determinants of dengue virus which increase host and vector transmissibility. The authors characterized a novel clinical isolate of Dengue-2 (TW2015) and found this virus is pathogenic in mice lacking type I and II interferon and STAT1 signaling. This analysis included increased mortality and higher virus virus replication in peripheral tissues as compared to the NGC strain. Next, the authors performed transmission studies with mosquitoes and mice and found that the virus can infect mice from mosquitoes and that the virus can be transmitted from mice to mosquitoes. Lastly, the authors identified that the PrM-E region is the main virulence determinant in mice. Overall, this is a nice study that includes analysis from clinical isolate characterization, infection of mice, infection of mosquitoes, and identification of virulence determinants. However, there are concerns about the rigor of several experiments (many of which it is unclear whether they were performed once or multiple times). Further, there were also some concerns about the growth kinetics of the chimeric viruses.

**Response:**

All the results included in the manuscript are representative and reproducible in multiple experiments. The growth kinetics of the chimeric viruses have been included in Fig 5C.

Part II – Major Issues: Key Experiments Required for Acceptance

Please use this section to detail the key new experiments or modifications of existing experiments that should be absolutely required to validate study conclusions.

Reviewer #1: (No Response)

Reviewer #2: Comments:

1. Figure 2- Was infectious virus recovered from the spleen and other organs with TW2015?

**Response:** As shown in Fig 2F, Infectious viruses could be recovered from the DENV-infected spleen, lung, and small intestine at 5 dpi. The virus titer was significantly higher in the TW2015-infected lungs than in the NGC-infected lungs. (Line 278-281)

2. Figure 3 A/B- These data are intersting, but are rather underpowered.

**Response:** The experiment was repeated with increased group size and a new data set was included in Fig 3A and 3B.

3. Figure 5- The authors mapped the virulence determinants using an infectious clone strategy. However, for several of the analyses, these data are rather preliminary and underpowered. Further, it is unclear whether these experiments were repeated or performed once.

**Response:** All the data included in the manuscript were representative of several tests. The group size in Fig 5E (originally Fig 5C) was 3 to 4. As one serum sample from 3 dpi was lost, the sample size was 2 in the original figure. We have replaced the serum titer data (3 dpi) with the data from 4 dpi.

4. Figure 5- Does the PrM/E chimeric virus have similar virus growth kinetics to the parental strains in Vero cells and other primary mouse/human cells? Does the PrM/E chimeric viruses alter cell infection (binding and entry)?

**Response:** Compared to the 16681 parental strain, the 16681/prM-E(2015) mutant had a slow virus growth kinetics in Vero 76 cells (Fig 5F). However, the TW2015 prM-E segment enhanced the virus binding affinity to human A549 lung epithelial cells (Fig 5G). (Line 386- 390)

5. Figure 7B- Again, some of these findings are underpowered.

**Response:** The infected mice were used as viremic blood source, so only few mice were used for the experiment in Fig7B and 7C. Evaluation of the mosquito infection rate with infected mice was performed on 2 and 3 dpi. The original Fig 7B and 7C only showed the mosquito infection rate with the mice at 2 dpi. The mosquito infection rate with the mice at 3 dpi was included in the revised Fig 7B and 7C.

Part III – Minor Issues: Editorial and Data Presentation Modifications

Please use this section for editorial suggestions as well as relatively minor modifications of existing data that would enhance clarity.

Reviewer #1: Abstract: it is unclear what the author’s are referring to when stating the TW2015 16681 virus. Is this a chimera between DENV-2 strain TW2015 and strain 16681?

**Response:** We apologize for the mistake. The sentence has been revised. (Line 51-52)

Summary: “dengue severe hemorrhagic fever” is not a clinical term.

**Response:** We apologize for the mistake. The sentence has been revised. (Line 58)

Introduction: the authors are remiss in not discussing the hypotheses and mechanisms that explain severe and fatal dengue. This would properly frame the rationale of the study.

**Response:** We thank the reviewer’s suggestion. A brief discussion has been included in the introduction section. (Line 89-95)

Methods: What is the passage history of all virus isolates used in this study and were they verified by sequencing? Similarly what was the passage history and sequence of the virus used to make the 16681 clone. Both NGC and 16681 are typically considered high passage viruses.

**Response:** We appreciate the reviewer’s comments. The TW2015 virus from patient serum was inoculated to Vero 76 cells and further propagated once in Vero 76 cell at Taiwan CDC. The TW2015 virus stock used in the study was within five passages　(Line 129-131). The 16681 parental virus and 16681 chimera viruses were generated from DNA plasmid (pTight-DENV2) or DNA fragment assembly. The insertion of the chimera viruses were all confirmed by sequencing. The 16681 chimera viruses used in the study were propagated in Vero 76 cells within six passages. (Line 217-21). The NGC is indeed a high passage virus strain.

Methods: please specify what “normal” housing conditions are for mosquito experiments, including temperature, relative humidity, and light cycle.

**Response:**

The mosquitoes for the related experiments were kept in an incubator maintained at 28 °C and 70% humidity and a 12 hour light/dark cycle with a 10% sucrose solution for 7 days. (Line 167-169).

Fig 1A: This does not show titers, this is a representative image of a plaque assay. Also, it is not exactly clear what the authors are trying to demonstrate here and in the first line of the results. Is this simply the titer after stock generation?

**Response:** We apologize for the confusion. Fig 1A was used to show that the plaque size produced from these virus strains was different. The figure has been revised and the quantification of plaque size was also included. (Line 242-245).

Fig1D: This panel is illegible. Is there a more informative, preferably quantitative way to show that the mice had DENV NS1 present.

**Response:** The NS1 detection was performed with immunoblotting as shown in Fig 1D.

Fig1E: why was only DENV-NGC used as a control for these experiments and not 16681?

**Response:** The NGC strain is a DENV-2 strain and has been used extensively in vitro and in vivo. Initially, we intended to identify virus strains that are highly pathogenic in mice and mosquitoes. 16681 strain was included later as the infectious clone of 16681 strain was helpful for the generation of the chimeric viruses. 16681 is low pathogenic in AGB6 mice even challenged with high dose (1×10^5^ pfu/mouse) as shown in Fig5D.

Fig 2: why was a different inoculum dose used for these experiments?

**Response:** When a low challenge dose (1×10^3^pfu/mouse) was used, the mice showed sympotums or died at various time points due to the variations in antiviral responses in animals. The advantage of a high challenge dose (1×10^5^ pfu/mouse) was to make the critical events occur at approximately the same time.

Fig 4F and G: For the transmission back to uninfected mice, how many mosquitoes were allowed to feed on the naive mice?

**Response:** As mentioned in the figure legend, the naïve mice were bitten by 6-9 infectious mosquitoes. (Line 353 an 683)

For the reverse genetics experiments, why was a clone based on the DENV strain 16681 strain used for chimera generation? It is fine if this was the most straightforward path but some justification for use of this strain, which up until this point was not included in any other experiment is warranted.

**Response:** We appreciate the reviewer’s comments. The generation of chimera genome requires high quality of PCR-amplified DNA fragments for in vitro ligation followed by DNA transfection for infectious virus production. The 16681 infectious clone pTight-DENV2 generated by Dr. Andrew Yueh previously has made this part of the work possible. Although the 16681 strain was not included in some parts of the experiments (Fig 1-4), the virulence of 16681 virus in AGB6 and mosquitoes was weaker than the TW2015 and NGC strains (Fig.5 and 7).

Fig 5B: Plaque size should be measured and compared using a statistical test.

**Response:** The plaque size for each virus strains was measured by Image J software and the statistical test was included. (Fig 5C; Line 375-378)

Discussion: the discussion could be improved to place the results in the appropriate context. Perhaps discussing previous DENV circulation in Taiwan and speculating about mechanisms that led to displacement of previous strains with this new strain.

**Response:** We thank the reviewer’s suggestion. DENV in Taiwan usually is transmitted from imported cases to indigenous cases in summer when the weather and rainfall are suitable for Aedes mosquitoes breeding. The outbreaks peak in fall and cease in winter due to the cold temperature and reduced rainfalls for the Aedes mosquito to live. DENV strains with high transmission potential and highly virulent in humans may cause large scale outbreaks easily in Taiwan. Hence, the transmission potential of each DENV genotypes may worth to characterize and put into consideration for dengue control.　(Line 441-448) Both D2Y98P and TW2015 strains belong to the Cosmopolitan genotype and the Cosmopolitan strains have become more and more dominant in the endemic countries. (Line 437-440)
